# The Lived Experiences of Bereaved Dementia Caregivers on the ALZConnected Peer Support Forum

**DOI:** 10.1093/geroni/igaf122.3860

**Published:** 2025-12-31

**Authors:** Mary Gemma O’Donnell, Zachary Baker, Sabrina Garcia-Arias

**Affiliations:** Arizona State University, Phoenix, Arizona, United States; Arizona State University, Phoenix, Arizona, United States; Arizona State University, Phoenix, Arizona, United States

## Abstract

Bereaved dementia caregivers face a 6–26% risk of complicated or prolonged grief, yet little is known about how they adapt after loss in the expanding landscape of online peer support. Digital forums provide spaces where caregivers share narratives, preserve bonds with the deceased, and seek meaning beyond the caregiving role, but these expressions remain largely undocumented in dementia bereavement research. This qualitative study examined how bereaved dementia caregivers express continuing bonds (CBs) that support adaptive grief in an online peer support context. Guided by Continuing Bonds Theory and a constructivist lens, the study employed a qualitative netnographic approach with reflexive thematic analysis to analyze posts from the ALZConnected caregiver forum. Data included 1,771 threads (2,012,195 words) from the “Supporting Those Who Have Lost Someone” discussion board, posted between 2011 and March 2023. Purposive sampling identified relevant narratives. Coding was conducted both deductively, using existing CB typologies, and inductively to capture novel caregiver-generated expressions. Six interpretive themes were identified: Living for Them Now, Love That Outlives Loss, Seeing Them in a New Light, Touchstones and Traces That Bond, Honoring Bonds in Shared Spaces, and Still Teaching, Still Giving. These themes illustrate how caregivers sustain ongoing connections that foster personal growth, meaning-making, and integration of loss. Findings offer practical relevance for clinicians, grief counselors, and support providers by identifying adaptive pathways that can be encouraged in bereavement interventions. This study advances theoretical understanding of CBs in dementia bereavement and underscores the role of online peer support in promoting adaptive grief.

